# Successful remission induction therapy with azacitidine and venetoclax for a treatment-naive elderly patient with ETP/myeloid mixed-phenotype acute leukemia: a case report

**DOI:** 10.3389/fonc.2025.1628767

**Published:** 2025-08-25

**Authors:** Kaikai Huang, Yanbin Pang

**Affiliations:** Department Hematopathology, Shenzhen Hospital of Southern Medical University, Shenzhen, China

**Keywords:** mixed-phenotype acute leukemia, venetoclax/azacitidine combination, COVID-19, elderly patient, nirmatrelvir/ritonavir, case report

## Abstract

**Background:**

Mixed-phenotype acute leukemia (MPAL) is a rare acute leukemia for which data are currently not available to guide therapy. It has a poor outcome, particularly in elderly patients.

**Case presentation:**

We report the successful use of venetoclax/azacitidine as treatment for a treatment-naive elderly patient with early T-cell precursor (ETP)/myeloid MPAL. Initial laboratory studies showed 62% blast cells and 32% lymphocytes on peripheral blood smear. Bone marrow aspiration showed two types of abnormal cell populations. Cytochemical staining showed that myeloperoxidase (MPO) was positive. Immunophenotyping with multicolor flow cytometry analysis showed two distinct populations of blasts with ETP acute lymphoblastic leukemia (ETP-ALL) and myeloid phenotype blasts, respectively. Molecular studies showed no abnormality of the fusion gene transcript. Missense mutation gene was found in genes including DNMT3A, JAK3, and NOTCH1 using next-generation DNA sequencing. Conventional karyotyping of this marrow aspirate revealed 46, XX[10]. She was diagnosed as having MPAL with two distinct blast lineages. Induction therapy was started using venetoclax/azacitidine. The patient developed COVID-19 on the second day of induction therapy. Consequently, the administration of subsequent doses of venetoclax/azacitidine for induction therapy was delayed, and nirmatrelvir/ritonavir was given as therapy for COVID-19. Fortunately, after 5 days of treatment with nirmatrelvir/ritonavir, the patient’s COVID-19 viral load became undetectable (nasopharyngeal swab negative) on 17 January 2023. During induction therapy, the patient was positive for COVID-19 twice but remained asymptomatic. Therefore, the induction treatment was not interrupted. She achieved complete remission with hematological recovery. She spontaneously developed anti-COVID-19 antibodies. The patient continued to receive treatment with venetoclax/azacitidine as planned. At the last follow-up in December 2023, the patient died after 11 months from the initiation of venetoclax/azacitidine because she gave up chemotherapy after 5 months.

**Conclusion:**

We report on an elderly patient with MPAL treated with venetoclax combined with azacitidine. This regimen successfully induced complete remission with no adverse side effects, and despite testing positive for COVID-19 multiple times during induction therapy, accompanied by mild dry cough but no radiographic evidence of pneumonia, the patient remained clinically stable.

## Introduction

Mixed-phenotype acute leukemia (MPAL) is an uncommon and heterogeneous acute leukemia characterized by blast cell expression of antigens from more than one lineage causing ambiguous classification (1, 2). MPAL is characterized by the detection of more than one lineage (myeloid lineage, B lineage, and T lineage), regardless of whether one or more population of blasts were found (3). It accounted for less than 5% of all acute leukemias; as such, data are currently not available to guide therapy with an absence of a clearly defined optimal treatment approach (4). The best therapy approach for adult MPAL remains unclear due to the absence of prospective randomized controlled clinical trials. Therefore, the MPAL treatment approach has been largely dependent on retrospective case series and case reports (5, 6).

Several studies have supported the idea that newly diagnosed MPAL could potentially benefit from acute lymphoblastic leukemia (ALL)-type or hybrid regimens [blending elements of acute myeloid leukemia (AML) and ALL regimens] over AML regimens (7, 8). This treatment may not be applied for adult patients, as the remission rate and overall survival of adult patients with MPAL were significantly lower than those of children (2). New targeted drugs, such as midostaurin and gilteritinib, and immunotherapy drugs, such as chimeric antigen receptor T cell and blinatumomab, can produce good therapeutic effects on certain target molecules or immune targets (9–11). However, there is still no effective treatment plan for elderly patients with MPAL who lack these targets. The novel regimen of venetoclax combined with hypomethylating agents (HMAs) has a promising synergistic therapeutic effect and a tolerable safety profile, which has been officially approved by NCCN Guidelines for newly diagnosed AML in adults who are 75 years or older or patients precluding intensive induction chemotherapy (12). Only a few patients with MPAL who were treated with venetoclax-based combinations have been described so far (13, 14).

COVID-19 has brought great challenges to the clinical treatment of acute leukemia. However, with the widespread use of the COVID-19 vaccine, antiviral therapy, and monoclonal antibodies in clinical practice, the prognosis of patients with ALL infected with COVID-19 has significantly improved (15). For some mild or asymptomatic infected individuals, anti-leukemia treatment may not lead to worsening of the condition (16). Some research results show that venetoclax/azacitidine could be safe and effective for patients with AML infected with COVID-19 (17). However, it was unclear whether elderly patients with MPAL infected by COVID-19 can benefit from the treatment of venetoclax combined with azacitidine. We present a rare case of MPAL with two separate lineages of blasts consisting of ETP and AML immunophenotypes, which has been infected with COVID-19 on the second day of treatment. Following antiviral treatment with nirmatrelvir/ritonavir, the patient’s COVID-19 nasopharyngeal swab tested negative on 17 January 2023. The patient achieved complete remission following treatment with venetoclax and azacitidine.

## Case description

### Clinical case

**Figure 1:**
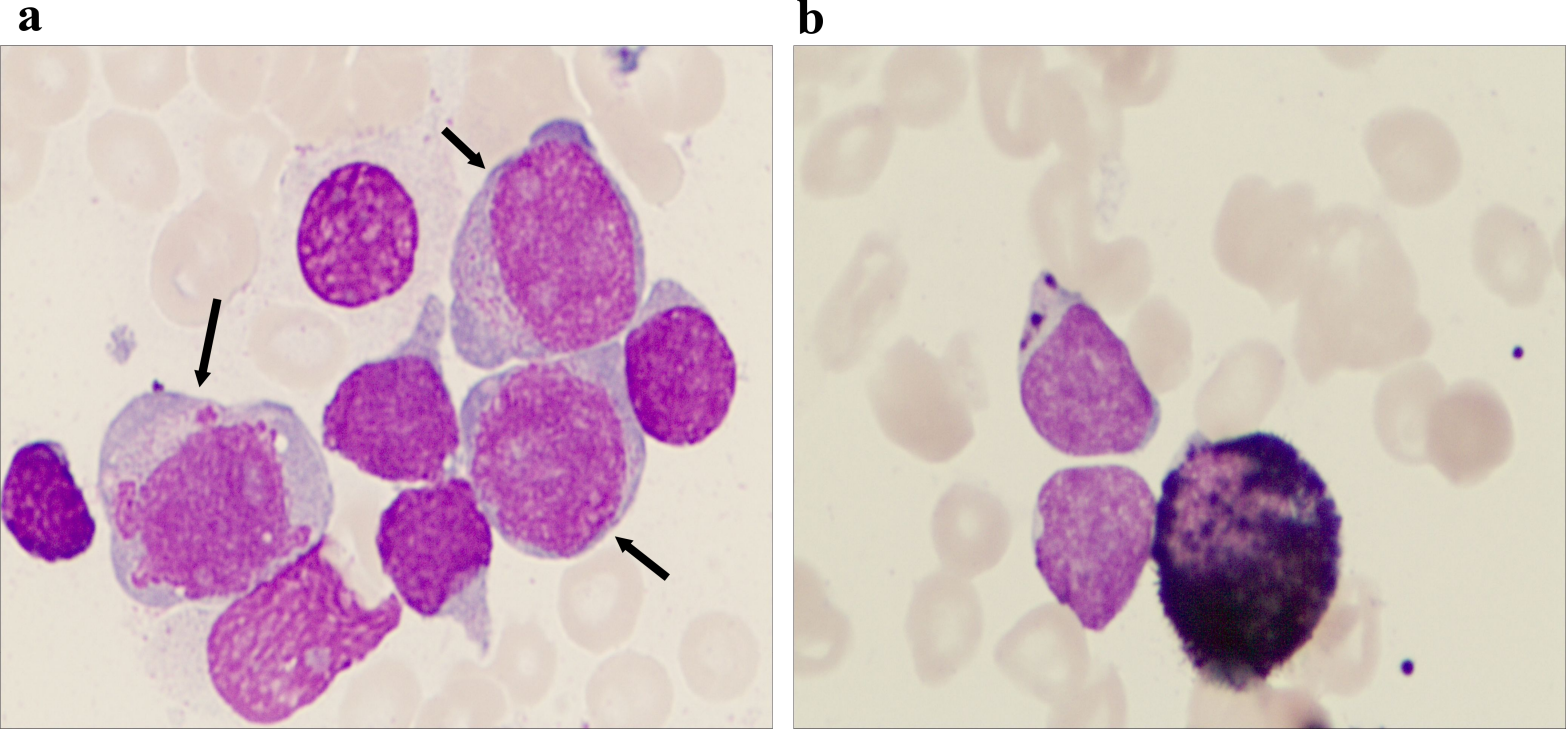
Morphological characteristics of the bone marrow aspirate. **(a)** The bone marrow picture shows that there are two groups of different-sized blast cell populations of different sizes in the bone marrow aspirate, which is a characteristic manifestation of mixed-phenotype acute leukemia (MPAL). The larger blast cells indicated by the arrow may represent the myeloid lineage, while the smaller blast cells may suggest the lymphoid lineage (Wright–Giemsa staining, 1,000× magnification). **(b)** Immunohistochemistry shows that myeloperoxidase (MPO) is positive in the original cells, confirming the presence of myeloid lineage components (MPO immunohistochemical staining, at 1,000× magnification).

**Figure 2:**
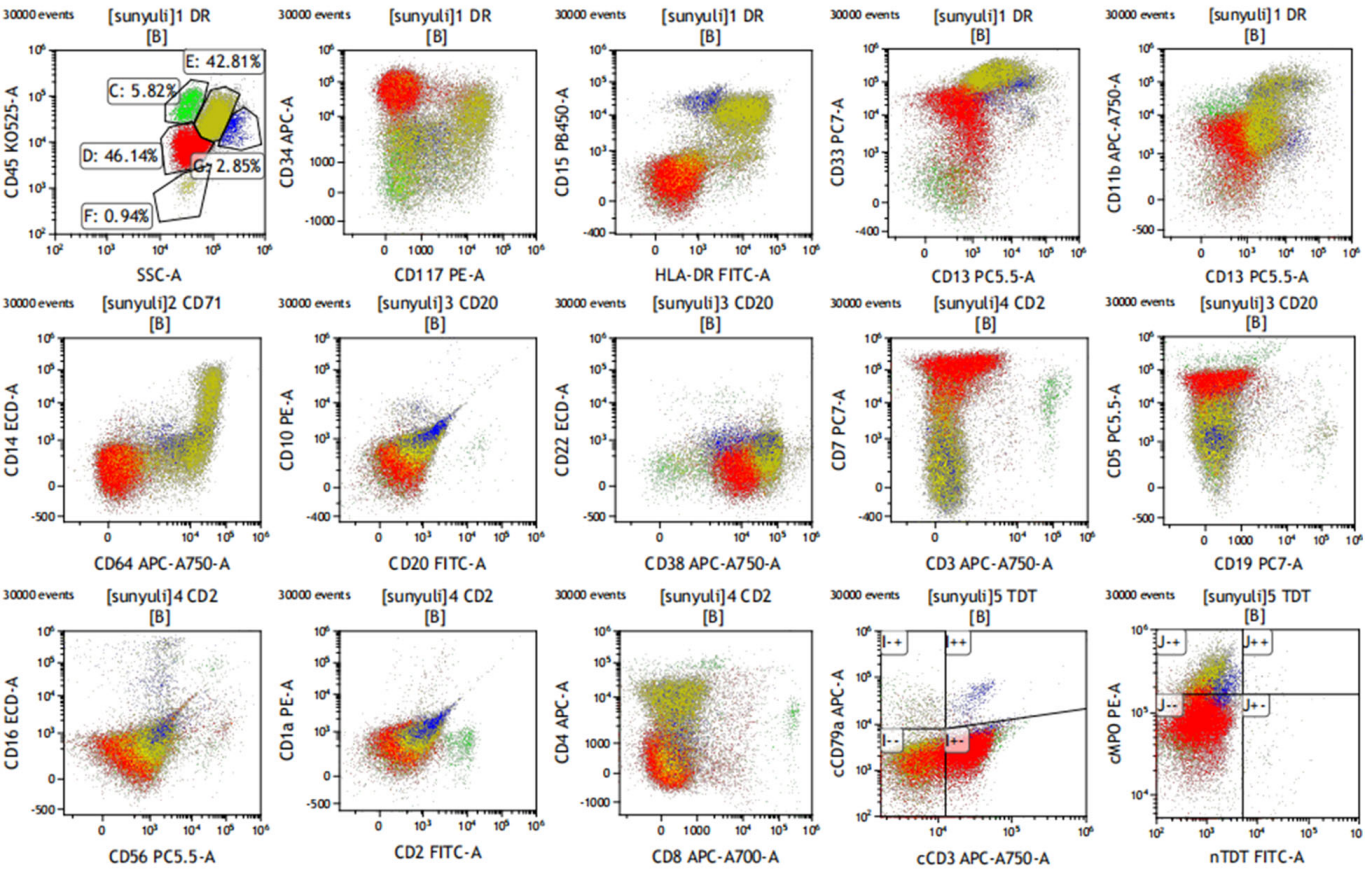
Immunophenotyping on bone marrow sample. Early T-cell precursor acute lymphoblastic leukemia immunophenotypes were characterized by a typical immunophenotype: cCD3, CD7, CD34, CD33, and CD5 (dim), and negative for sCD3, CD1a, CD8, and the other myeloid and B-lineage antigens. The other population consisted of promonocyte-like cells characterized by the co-expression of CD33 (bright), HLA-DR, CD64, CD14, CD36, CD15, CD117 (dim), cMPO (dim), and CD4 (dim).

A 73-year-old woman was admitted to the Department of Hematology of the Shenzhen Hospital of Southern Medical University in January 2023 with a 10-day history of bleeding gums. Physical examination showed mild pallor and scattered superficial lymphadenopathy. Routine laboratory tests, including peripheral blood count and smears, revealed leukocytosis with anemia and thrombocytopenia (a white blood cell count of 10.23 × 10^9^/L, a hemoglobin level of 93 g/L, and a thrombocytopenia level of 30 × 10^9^/L). There were 62% blast cells in the peripheral blood smear. Morphological analysis of the bone marrow (BM) aspirate suggested acute leukemia with approximately 59% of blast cells and, interestingly, approximately 20% of promonocytes (**Figure 1**). These blast cells were positive for myeloperoxidase (**Figure 1**). Immunophenotyping was performed on the leukemic cells from the BM aspirate by multicolor flow cytometry using the standard stain lyse–wash technique. This confirmed the expression of two distinct blast populations, which were noted on the CD45 vs. SSC plot. One population was characterized by a typical early T-cell precursor acute lymphoblastic leukemia immunophenotype: cCD3, CD7, CD34, CD33, and CD5 (dim), and negative for sCD3, CD2, CD4, CD1a, CD8, CD13, CD11b, cMPO, CD79a, CD19, CD10, CD117, and other myeloid and B-lineage antigens. The other population consisted of promonocyte-like cells characterized by the co-expression of CD33 (bright), HLA-DR, CD64, CD14, CD36, CD15, CD117 (dim), cMPO (dim), and CD4 (dim), and negative for CD19, CD3, CD79a, nTdT, CD13, and other myeloid and lymphoid antigens (**Figure 2**). Next-generation sequencing (NGS) of the BM identified missense mutations in DNMT3A (exon 16, VAF 83%), JAK3 (exon 19, VAF 44%), and NOTCH1 (exon 26, with two distinct mutations of VAF 6% and 32%, respectively). Conventional karyotyping of this marrow aspirate revealed a normal chromosome karyotype. In this case, no abnormalities were found by polymerase chain reaction and fluorescence *in situ* hybridization, such as PML/RARa, BCR/ABL, AML1/ETO, CBFb/MYH11, and MLL. Therefore, this case was ultimately diagnosed as MPAL (T+My)-NOS by combining morphology and flow immunophenotype.

**Figure 3:**
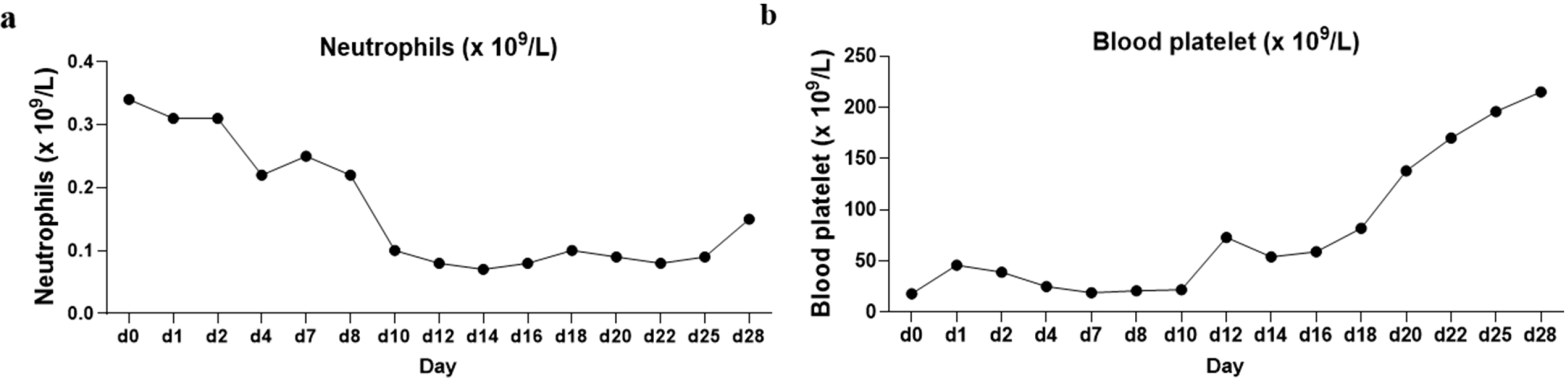
Trends of neutrophil and platelet changes during patient induction therapy. **(a)** Neutrophil trends. The temporal changes in neutrophil levels during the course of induction therapy. The time-based *x*-axis represents the duration of treatment, and the *y*-axis indicates neutrophil counts. The trend reveals fluctuations typical of such therapies. **(b)** Platelet trends. The corresponding variations in platelet counts over the same treatment period.

**Figure 4:**
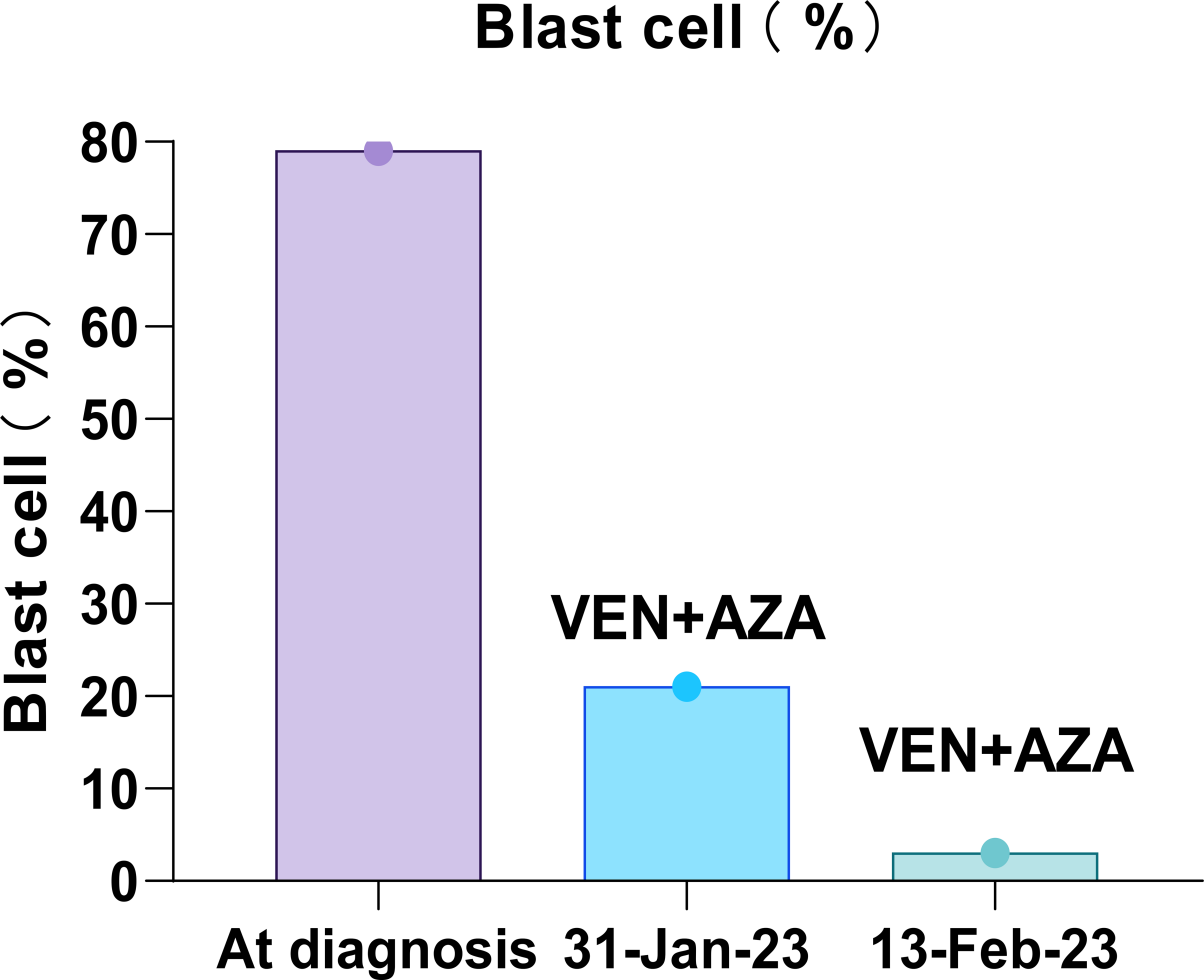
Trend of bone marrow blast cell dynamics during patient induction therapy. The time-based x-axis spanned the duration of the treatment period from 11 January 2023 to 20 February 2023, and the *y*-axis indicates the percentage of blast cells. The venetoclax and azacitidine regimen illustrates a significant initial reduction in blast cells, indicating morphological remission.

**Figure 5:**
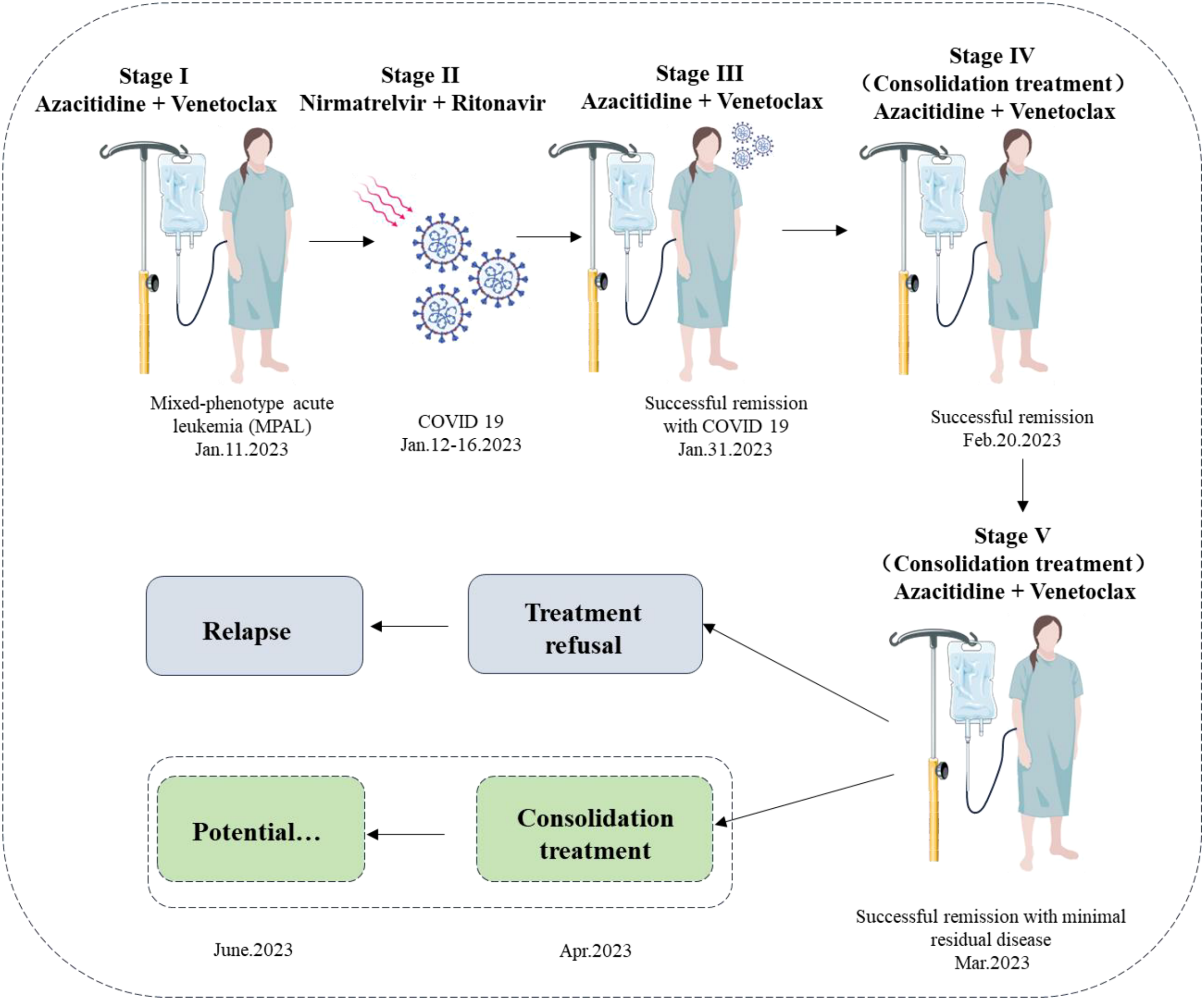
Timeline of the patient’s clinical course and treatment regimen. The schematic diagram illustrates the treatment timeline and clinical progression of the patient with mixed-phenotype acute leukemia (MPAL) diagnosed on 11 January 2023. Stage I (11 January 2023) depicts the initiation of induction therapy with azacitidine and venetoclax. Stage II (12–16 January 2023) shows the interruption due to a COVID-19 infection, treated with nirmatrelvir/ritonavir, with viral load becoming undetectable by 17 January 2023. Stage III (17–30 January 2023) represents the resumption and continuation of azacitidine and venetoclax, achieving successful remission by 31 January 2023. Stage IV (consolidation treatment) indicates ongoing azacitidine and venetoclax therapy from 20 February 2023, maintaining successful remission with minimal residual disease. Stage V (consolidation treatment) suggests potential continued therapy. Unfortunately, owing to treatment refusal, the patient relapsed on June 2023. The dashed box shows a hypothesis: if a potential consolidation treatment was performed in April 2023, she could have sustained remission.

She was treated with a combination of venetoclax (100 mg on day 1, 200 mg on day 2, and 400 mg on days 3–28) and azacitidine (75 mg/m^2^ on days 1–7) on 11 January 2023. On 12 January 2023, she developed a fever, and although she denied any respiratory symptoms, a combined nasal and pharyngeal swab for COVID-19 RNA was positive. Given her active COVID-19 infection, she received nirmatrelvir/ritonavir on 12 January 2023. Concurrently, venetoclax/azacitidine was temporarily discontinued. On 17 January 2023, the patient’s COVID-19 nasopharyngeal swab tested negative. She resumed treatment with venetoclax and azacitidine, with close monitoring of laboratory and clinical parameters for signs of tumor lysis syndrome. Fortunately, the patient showed no signs of tumor lysis during induction therapy. However, the patient experienced severe neutropenia (<0.5×10^9^/L) (**Figure 3**) and thrombocytopenia (<50×10^9^/L) for 12 days (**Figure 3**). Although the patient was positive for COVID-19 on 26 January 2023 and on February 7, 2023, respectively, the patient had no fever, dyspepsia, and other COVID-19-related symptoms. The patient was considered to have been infected with COVID-19 but was asymptomatic and, thus, continued to be treated with venetoclax. On 13 February 2023, the patient’s platelet count returned to normal; moreover, BM examination revealed that blast cell ratio was 3% (**Figure 4**). Thanks to the effective treatment regimens, the patient finally achieved complete remission with incomplete count recovery (CRi). She received one more cycle of venetoclax/azacitidine therapy. However, the detection of 4% ETP cells in March 2023 indicates persistent minimal residual disease (MRD). Following the completion of the induction regimen [venetoclax + azacitidine (VA) combined with vincristine 2 mg on days 8 and 22, and prednisone 30 mg from days 8 to 21 (VP)], the patient discontinued therapy in April 2023. Approximately 2 months later, on 14 June 2023, morphological relapse was confirmed, with flow cytometry showing 40% ETP cells and 11% primitive monocytic cells, consistent with the T/myeloid MPAL phenotype observed at diagnosis. At the last follow-up in December 2023, the patient died after 11 months from the initiation of venetoclax/azacitidine because of infection and bleeding (**Figure 5**).

## Discussion

While substantial progress has been made and robust data exist informing on the treatment of AML, data regarding MPAL treatment are not available. The immunophenotype of MPAL may guide the choice of induction therapy. One study found that the expressing CD19 (which included a positive result or a partial positive result in at least one blast population in cases with MPAL in children) benefited from ALL-type therapy, and the 5-year event-free survival (EFS) rate was 83% ± 5.3%, while the 5-year EFS rate of AML and mixed-type (blending elements of AML and ALL regimens) therapy was 0% ± 0% and 28% ± 14%, respectively (both *p* < 0.0001) (18). Patients with other lymphoid phenotypes also benefit from ALL-type therapy (18). The current literature suggests that newly diagnosed MPAL could potentially benefit from ALL-type regimens over AML regimens (7, 8). However, adult patients benefit from the ALL approach significantly less than children, as shown by the remission rate of adults using the ALL regimen (51.5%), which is significantly lower than that of children (77.8%, *p* < 0.05). Median survival was 11 months for adults and 3-year overall survival rate was only 16.4%, while the corresponding values for children are 139 months and 39.5%, respectively (2). Most importantly, elderly patients also had COVID-19 infection. The traditional induction treatment scheme may aggravate the infection of patients and significantly increase the risk of death (19). Therefore, traditional chemotherapy regimens for adult MPAL continue to face challenges.

Venetoclax, an oral selective inhibitor of the anti-apoptotic protein B-cell leukemia/lymphoma 2 (BCL-2), in combination with HMAs has worked effectively in preclinical models, suggesting that this is an effective treatment for AML (20). Venetoclax, as a single agent in a phase II study for patients with relapsed/refractory (R/R) AML, has been shown to have an overall response rate of 19% (21). Subsequently, venetoclax and HMA combined therapy showed positive anticancer activity and was found to be safe in older patients with AML for whom conventional chemotherapy was not suitable (22). Moreover, venetoclax may also be a novel therapeutic strategy for early T-cell acute lymphoblastic leukemia (23). The choice of AZA+VEN was informed by emerging evidence supporting the efficacy of venetoclax in ETP-ALL, which is highly dependent on the anti-apoptotic protein BCL-2 for survival, unlike more mature T-ALL subtypes that rely on BCL-XL. Specifically, Numan et al. (24) reported the first clinical response to venetoclax in ETP-ALL, demonstrating improved treatment outcomes when venetoclax was combined with conventional chemotherapy. Additionally, the synergistic effect of venetoclax with HMAs like azacitidine has shown promise in AML and leukemias of ambiguous lineage, providing a strong scientific basis for its use in MPAL (21, 25). More importantly, the patient’s BM NGS revealed missense mutations in DNMT3A, JAK3, and NOTCH1. DNMT3A mutations are frequently observed in myeloid malignancies and may suggest a clonal myeloid origin in MPAL, potentially contributing to a poorer prognosis due to their association with epigenetic dysregulation (26). In MPAL, abnormal DNA methylation plays a critical role in disease pathogenesis, making DNMT3A a potential therapeutic target for HMAs like azacitidine (27). JAK3 mutations, less common in MPAL, are associated with lymphoid malignancies and may indicate aberrant signaling in the T-cell lineage. NOTCH1 mutations are prevalent in T-ALL, including ETP-ALL, and are known to drive leukemogenesis, but their presence in MPAL often correlates with poor response to conventional chemotherapy (26). These data provide the clinical basis for MPAL (T cell/myeloid) treatment with combined venetoclax and HMAs. In addition, induction therapy with venetoclax and HMAs may not increase the risk of death of acute leukemia patients with COVID-19. Ghandili et al.’s (17) results showed that among the six patients who received venetoclax + azacytidine treatment, three were newly diagnosed patients with AML, and three were relapsed refractory patients. Two were newly diagnosed patients and one relapsed refractory patient obtained CRi. Among the three initially treated patients, two developed severe acute respiratory distress syndrome (ARDS) after induction treatment with azacitidine/venetoclax, and no patient died due to induction treatment. The remaining three patients received only one course of induction therapy but did not develop ARDS, while one newly diagnosed AML patient who received standard regimen treatment died due to COVID-19 infection. Other studies have also shown that the risk of death may increase in patients with acute leukemia complicated with COVID-19 infection who received the standard treatment scheme. Núñez-Torrón et al. (28) conducted a retrospective analysis of four newly diagnosed COVID-19-infected patients with AML using standard induction therapy and found that three patients died due to treatment-related infections. Based on the above reasons, we choose azacytidine combined with venetoclax as the treatment plan for this patient with MPAL. Similar to other researchers, this patient did not experience severe infection and tumor lysis syndrome, although she also experienced severe agranulocytosis and thrombocytopenia after treatment with venetoclax/azacytidine (28). However, in this case, the combination of DNMT3A and NOTCH1 mutations likely contributed to the aggressive disease course and rapid relapse observed after treatment discontinuation in April 2023, despite initial morphological remission. The persistence of 4% ETP cells in March 2023 suggests residual disease driven by these mutations. Emerging evidence suggests that histone deacetylase inhibitors (e.g., chidamide) could enhance treatment efficacy in patients with MPAL with DNMT3A and NOTCH1 mutations by targeting epigenetic and transcriptional dysregulation (29).

Second, unlike other reported cases, this patient tested positive for COVID-19 at the start of treatment and again twice during induction therapy, with mild dry cough but no radiographic evidence of pneumonia, suggesting residual viral shedding rather than distinct reinfections. One might ask, how are COVID-19-positive patients treated during induction therapy? This patient was treated with nirmatrelvir/ritonavir at the initial stage of infection, but induction therapy was not delayed after a negative COVID-19 result. During induction treatment, the patient developed COVID-19 infection with no respiratory symptoms. The induction treatment was continued under close monitoring, although at the beginning of the prevalence of COVID-19, the general consensus of experts is to start induction treatment 14 days after testing negative for COVID-19 (16). However, more research results showed that the death of patients with AML infected with COVID-19 was related to developing severe or critical infection, and induction treatment does not increase the risk of death among mild COVID-19-infected patients with AML (16). With the widely available COVID-19 vaccines and the progress of antiviral and supportive treatment, the incidence of severe or critical cases of COVID-19 infection in patients with AML has significantly decreased; thus, this may be an important factor for the significant decrease in the time from diagnosis to induction therapy in patients with AML. The current median time was 1 week, and only 8.7% of patients have delayed treatment for more than 1 month (16). The mortality rate within 30 days of early initiation of induction treatment of AML was only 9.3%, and the mortality rate does not seem to increase (21). Interrupting treatment after induction chemotherapy was a high-risk factor for death. At a median follow-up of 266.5 days, the overall survival rate of patients with interrupted treatment was only 6%, and the overall survival rate of patients without delay or interruption of treatment was 64% (16). Therefore, during the re-induction treatment, although the patient was infected with COVID-19, because there was no clinical evidence that the patient might develop severe or critical infection, the management of this elderly MPAL patient was complicated by concurrent COVID-19, highlighting the need for careful monitoring and antiviral therapy to mitigate infection-related risks during leukemia treatment.

Lastly, one of the most frequently reported hematologic adverse events (grade 3 or higher) of azacitidine–venetoclax treatment was neutropenia and febrile neutropenia (22). The patient developed persistent neutropenia during induction therapy. However, she did not experience severe febrile neutropenia. This could be related to the ability of venetoclax to restore the immune function of lymphocytes. It has been demonstrated that venetoclax combined with G protein synergistically activated p38 MAPK, which induces mitochondrial-related cell apoptosis and gasdermin E (GSDME)-dependent myeloptosis/myeloid leukemia 1 (MCL-1) axis. More importantly, leukemic pyroptosis enhanced CD8^+^ T-cell immune function by the release of interleukin-1β/18 into the tumor microenvironment (30).

## Conclusion

Although further studies are required in this setting, the new venetoclax–HMA therapy has shown promising efficacy and tolerable safety in the initial treatment of MPAL, which can be considered as a treatment option for patients with MPAL.

## Data Availability

The original contributions presented in the study are included in the article/supplementary material. Further inquiries can be directed to the corresponding author.
